# Does FTO have a paradoxical effect in fetal life?

**DOI:** 10.1186/s12863-014-0145-0

**Published:** 2014-12-24

**Authors:** Olivier S Descamps, Eric Tarantino, Pierre-Francois Guilmot

**Affiliations:** Center for Medical Research at Jolimont, 159 Rue Ferrer, B-7100 Haine Saint-Paul, Belgium; Department of Internal Medicine, Centre Hospitalier Jolimont-Lobbes, 159 Rue Ferrer, B-7100 Haine Saint-Paul, Belgium; Department of Obstetrics and Gynecology, Centre Hospitalier Jolimont-Lobbes, 159 Rue Ferrer, B-7100 Haine Saint-Paul, Belgium

**Keywords:** Obesity, FTO, Newborn, Mother, Birth weight, Adiposity

## Abstract

**Background:**

Low weight at birth is associated with obesity in later life. One hypothesis to explain such an association is that genetic variants that increase the risk of obesity also reduce fetal weight. Recently, obesity in adults was found to be associated with common variants of the fat mass and obesity-associated (FTO) gene. We examined the association between FTO polymorphisms and birth weight in a singleton, full-term birth cohort of 494 newborn-mother pairs without any complications.

**Results:**

The risk alleles for obesity (“A” allele for the rs9939609 FTO variant and “G” allele for the rs9930506 FTO variant) were associated with low weight at birth. The mean differences per risk allele were −79 g (95% CI: −129 to −30; p = 0.002) for rs9939609 and −84 g (95% CI: −131 to −36; P < 0.001) for rs9930506. The level of association remained statistically significant after adjustment for the maternal risk allele and for variables usually associated with birth weight (−50 g, 95% CI: −99 to 0; p = 0.05 for rs9939609 and −48 g, 95% CI: −100 to 0; p = 0.05 for rs9930506). In the follow-up, the allelic difference in weight was attenuated over time.

**Conclusions:**

The FTO variants that confer a predisposition to obesity later in life appear to be associated with low weight at birth. This finding favors the hypothesis of a common genetic denominator that predisposes to a low weight at birth and obesity in adults.

## Background

Low birth weight is associated with an increased prevalence of obesity and insulin resistance syndrome in adult life, leading to an increased risk of type 2 diabetes, hypertension, and cardiovascular disease [1-5]. Although the mechanisms for this association are unknown, researchers have proposed that it reflects fetal programming *in utero* in response to maternal malnutrition during pregnancy [2,6,7]. An alternative hypothesis [8] is that genetic variants that increase the risk of disease also reduce fetal weight.

Several independent, genome-wide, association studies have recently identified a strong correlation between fat mass and obesity-associated (FTO) polymorphisms and obesity-related parameters (body mass index [BMI], total body weight, and hip circumference) in adults and children [9-11]. In childhood, although the known FTO risk alleles for obesity are associated with an increased BMI in the postnatal period [12], they have no effect on birth weight. This conclusion was drawn from large cohorts that were not controlled for situations affecting birth weight, such as gestational diabetes [13] or other gestational complications.

In the present study, we investigated the effect of two common FTO polymorphisms on birth weight in a singleton, full-term birth cohort without any maternal or newborn complications.

## Methods

### Study design

The mothers and their newborns were consecutively recruited in the maternity ward during 2008 and 2009. The nurses of the maternal unit collected blood samples (cord blood) and recorded maternal (at entry) and newborn (at delivery) anthropometric parameters. Birth weight was measured to the nearest 1 g using a digital baby scale Seca model 727 (Seca Belgium, Zwijndrecht), which includes a special damping system that allows for precise weighing, even if the newborn is restless. Two trained study nurses extracted data (parity, weight measured at the first antenatal clinic, and gestational age estimated from early obstetric ultrasound) from the obstetric medical records and interviewed the mothers to obtain data on smoking status and the genealogical tree (four generations).

The selection criteria of the mother–newborn pairs were a Caucasian origin, eutocic delivery with cephalic presentation between the beginning of the 37th week and the end of the 41st week, singleton live birth, no maternal use of alcohol or illicit substances, no gestational complications, no diabetes, no congenital malformations or perinatal problems, and an Apgar score of ≥ 7 during the 1st minute and ≥ 9 by the 5th minute. Twenty-nine of the original mother–newborn pairs who were recruited by the nurses were excluded because of non-compliance with one or more inclusion criteria, difficulty of genotyping, or important missing data.

For infants participating in the follow-up program of our hospital, body weight at 3, 6, 9, and 12 months was collected from the pediatric medical records. For the other children, we attempted to obtain data 1 year later by phone for the most recent weight of the child. The study protocol was approved by the Ethical Committee of the Hospital of Jolimont (ECO22) and informed written consent was obtained from all mothers before participation.

### Genotyping

We examined two single nucleotide polymorphisms (SNPs) of FTO (SNPs with the A risk allele for rs9939609 and the G allele for rs9930506) that were previously shown to be strongly associated with obesity-related parameters in large European populations [9,14]. We were particularly interested in the G allele for rs9930506 because it shows an even stronger association in Sardinian and Italian populations [15,16] and because some infants born in our hospital are partially of Italian descent (third or fourth generation born in Belgium). Genotyping of rs9930506 and rs9939609 was performed by Restriction Fragment Length Polymorphism-Polymerase Chain Reaction and Allele-Specific Oligonucleotide-Polymerase Chain Reaction, respectively, with a success rate greater than 97.5%.

### Estimation of sample size

Sample size was estimated for testing the primary hypothesis that birth weight is different between newborns with different genotypes (BMI was not used because measurement of length is less reliable in newborns). Based on the weights obtained in the first 100 newborns (mean ± standard deviation: 3284 ± 347 g), the minimum size for each group that was required to detect a 5% difference (a priori considered as relevant) was estimated as 70, for a two-sided significance level of 0.05 and a statistical power of 80% [17]. Based on the minor allele (the “risk allele”) frequency obtained in the first 100 newborns (approximately 40% for both SNPs) and assuming Hardy–Weinberg equilibrium, we set our target size to a minimum of 447 mother–newborn pairs.

### Statistical analysis

The analyses focused on birth weight. We examined associations separately for newborn and maternal genotypes, and then mutually adjusted one for the other, as well as for other non-genetic factors using multiple linear regression analysis (SPSS for Windows, version 8.0). Adjustment for maternal genotype is important because of the strong association between maternal and newborn genotypes and our aim to establish whether any associations are primarily driven by newborn genetic variants independent of maternal genotypes. Other non-genetic factors that are known to affect birth weight were examined, including racial/ethnic origin (all subjects were Caucasians but some were of Italian origin), sex, gestational age, parity, cigarette smoking, maternal BMI, and weight gain during pregnancy. In all analyses, a per-allele additive genetic model was examined.

## Results

The characteristics of the newborns and their mothers are shown in Table 1. The risk allele frequencies were 0.41 for rs9939609 and 0.43 for rs9930506, similar to those reported in European cohorts (0.40 [9] and 0.44 [15]), in Flanders (0.41 for rs9939609 [18]), in Italians (0.48 and 0.50 [16]), and in Sardinians (0.46 and 0.46 [15]) (Table 2). There was no evidence of departure from the Hardy–Weinberg equilibrium (Table 2) or an association with the number of Italian great-grandparents (data not shown).

**Table 1:** **Characteristics of the newborns and their mothers**

**Characteristics**		**Eligible data**	**Values**
Boys, N(%)		494	243(49%)
Weight, g		494	3251,8 ± 383,6
Lenght, cm		494	50,1 ± 2,0
Head circumference, cm		494	34,0 ± 1,4
BMI, kg/m^2^		494	13,0 ± 1,1
Ge stational age, weeks		494	39,1 ± 1,2
Mothers characteristics		494	
	Nullipara, N(%)	494	191(39%)
	Smoking during pregnancy, N(%)	494	101(20%)
	Age, years	494	28,7 ± 5,4
	Height, cm	494	164,7 ± 6,1
	Weight before delivery, kg	494	80,0 ± 14,8
	BMI before delivery, kg/m^2^	494	29,45 ± 5,02
	Weight at pregnancy diagnosis, kg*	486	67,2 ± 15,4
	Calculated gain in pregnancy, kg**	486	12,8915,4
	Weight after delivery, kg	450	73,0914,6
	Calculated loss after delivery, kg*	450	-7,0 ± 2,2
Genealogical data		485	
	0 Italian Great-grandparents, N(%)		278(57%)
	1-2 Italian Great-grandparents, N(%)		62(13%)
	3-4 Italian Great-grandparents, N(%)		73(15%)
	5-6 Italian Great-grandparents, N(%)		27(6%)
	7-8 Italian Great-grandparents, N(%)		45(9%)

*self reported or unstandardized.

**calculated from self reported or unstandardized data on weight at pregnancy diagnose.

**Table 2:** **Frequencies of FTO genotypes and alleles**

**FTO rs9939609**			**Newborns with TT genotype**	**Newborns with TA genotype**	**Newborns with AA genotype**	HWE P value*
Total		494	167(34%)	253(51%)	74(15%)	0.41
Maternal genotype						
	TT	167(32%)	95(57%)	72(28%)		
	TA	248(50%)	72(43%)	133(53%)	43(58%)	0.17
	AA	79(16%)		48(19%)	31(42%)	
**FTO rs9930506**			**Newborns with AA genotype**	**Newborns with AG genotype**	**Newborns with GG genotype**	***HWE P value***
Total		494	165(33%)	236(48%)	93(19%)	0.57
Maternal genotype						
	AA	155(31%)	93(56%)	62(26%)	-	
	AG	249(50%)	72(44%)	124(53%)	53(57%)	0.60
	GG	90(18%)	-	50(21%)	40(43%)	

*Chi-square test.

HWE: Hardy Weinberg equillibrium.

### Association of newborn FTO and birth weight

For both SNPs, newborn homozygotes for the risk allele weighed significantly less than newborn heterozygotes, and weighed even less than the homozygotes for the non-risk allele, with a clear gene-dose effect (Table 3). The newborn risk alleles were associated with a lower birth weight, with a mean difference per risk allele of −78 g (95% confidence interval [CI]: −128 to −28; p = 0.002) for rs9939609 and −83 g (95% CI: −131 to −36; p = 0.0006) for rs9930506. The level of association did not change when we adjusted for the maternal risk allele (Table 3). Low birth weight was associated with maternal risk alleles in univariate analyses (Table 4), but this association may have been attributed to the offspring’s FTO gene because it was not more significant after adjustment for newborn risk alleles.

**Table 3:** **Associations of birth weight and newborn FTO genotypes or alleles**

		**Exposure = Newborn FTO SNP**					
		**Newborn weight (g)**			**FTO rs9930506 SNP**		
		**N**	**Mean ± SD(g)**	P value vs 1°group*	**N**	**Mean ± SD(g)**	P value vs 1°group*
Genotypes							
	Homozygous for non rish allele	167	3314 ± 378		165	3326 ± 386	
	Heterozygous	253	3238 ± 386	0,02	236	3236 ± 394	0,02
	Homozygous for risk allele	74	3157 ± 366	0,001	93	3161 ± 329	0,0006
		**Mean difference (g) per newborn risk-allele for rs9939609**			**Mean difference (g) per newborn risk-allele for rs9930506**		
		**Mean (95% CI)**		**P value**	**Mean (g) (95% CI)**		**P value**
Various adjustments							
	Unadjusted	-78(-128; -28)		0,002	-83(-131; -36)		0,0006
	Adjusted for maternal allele	-73(-130; -16)		0,09	-76(-132; -21)		0,007
	Adjusted for maternal allele and other variables**	-56(-107; -5)		0,03	-55(-105; -6)		0,03
	Adjusted also for maternal BMI	-50(-99; 0)		0,05	-48(-100; 0)		0,05
	Adjusted also for Adjusted also for weight gain	-48(-98: +2)		0,06	-47(-95;+1)		0,06

*Students T test.

**Adjusted for sex, gestational age, panty and current smoking during pregnancy.

**Table 4:** **Associations of birth weight and maternal FTO genotypes and alleles**

		**Exposure = Maternal FTO SNP**					
		**FTO rs9939609 SNP**			**FTOrs9930506 SNP**		
		**Newborn weight (g)**			**Newborn weight (g)**		
		**N**	**Mean ± SD (g)**	P value vs 1°group*	**N**	**Mean ± SD (g)**	P value vs 1°group*
Genotypes							
	Homozygous for non risk allele	155	3312 ± 383		155	3312 ± 383	
	Heterozygous	249	3227 ± 381	0,03		3227 ± 381	0,03
	Homozygous for risk allele	90	3216 ± 383	0,06		3216 ± 383	0,06
		**Mean difference (g) per offspring risk-allele for rs9939609**			**Mean difference (g) per offspring risk-allele for rs9939609**		
		**Mean(95%CI)**		**P value**	**Mean(95%CI)**		**P value**
Various adjustments							
	Unadjusted	-53(-102; -4)		0,03	-45(-94; 5)		0,08
	adjusted for newborn allele	-13(-70; 43)		0,65	-10(-66; 47)		0,20
	adjusted for newborn allele and other variables**	-10(-61; 41)		0,70	-15(-66; 36)		0,57

*Student’s T test.

**Adjusted for sex, gestational age, panty and current smoking during pregnancy.

Birth weight was correlated with sex (r = 0.15, p = 0.001), gestational age (r = 0.33, p < 0,001), parity (r = −0.09, p = 0.06), cigarette smoking (r = −0.26, p < 0.001), and maternal BMI (r = 0.17, p < 0.001), but not with Italian origin of the grandparents (r = −0.06, p = 0.21). There was only a trend toward statistical significance with weight gain during pregnancy (r = 0.07, p = 0.13). None of these factors was significantly associated with the risk alleles. In particular, maternal BMI was not significantly associated with newborn risk alleles, but it was significantly associated with maternal risk alleles (p = 0.03 for maternal rs9930506 and p = 0.01 for the maternal rs9939609). Additionally, maternal risk alleles were associated with newborn risk alleles. Adjustment for factors that were significantly (p < 0.05) associated with birth weight (sex, gestational age, parity, current smoking during pregnancy, and maternal BMI) only slightly decreased the strength of the associations between newborn risk alleles and birth weight, without affecting the statistical significance (Table 3). These associations were not modified by inclusion of weight gain during pregnancy.

### Comparison of weight between the genotypes in childhood

There was no difference in weight between the genotypes 3 months after birth in the subsamples of children whose weight we could directly measure. There was also no difference in weight between the genotypes 1 year after birth in the 371 infants for whom we could obtain information by phone (Table 5).

**Table 5:** **Change in weight of infants according to genotypes**

**FTO rs9939609**		**Newborns with TT genotype**		**Newborns with TA genotype**		**Newborns with AA genotype**	Statistic (P values)*	
	**N**	**mean (g) SD**	**N**	**mean (g) SD**	**N**	**mean (g) SD**	**G2 vs G1**	**G3 vs G1**
At birth	167	3314 ± 378	253	3238 ± 386	74	3157 ± 366	0,05	0,00
3 months	31	5358 ± 404	60	5467 ± 627	13	5271 ± 593	0,38	0,58
6 months	25	7199 ± 494	54	7374 ± 635	13	6989 ± 618	0,23	0,26
9 months	31	9242 ± 491	55	9058 ± 779	14	9058 ± 352	0,24	0,21
1 year	127	10706 ± 753	190	10781 ± 917	54	10738 ± 550	0,44	0,78
**FTO rs9930506**		**Newborns with AA genotype**		**Newborns with AG genotype**		**Newborns with GG genotype**	**Statistic (P values)**	
	**N**	**mean (g) SD**	**N**	**mean (g) SD**	**N**	**mean (g) SD**	**G2 vs G1**	**G3 vs G1**
At birth	165	3326 ± 386	236	3236 ± 394	93	3161 ± 329	0,00	0,00
3 months	30	5325 ± 386	56	5496 ± 648	18	5283 ± 545	0,19	0,76
6 months	25	7023 ± 358	50	7343 ± 685	17	7164 ± 656	0,34	0,80
9 months	30	9158 ± 517	51	9059 ± 787	19	9197 ± 423	0,54	0,78
1 year	126	10798 ± 761	173	10635 ± 893	72	10937 ± 675	0,10	0,20

*Student’s T test.

## Discussion

In the present study, FTO variants that confer a predisposition to obesity later in life appeared to be associated with a low weight at birth. This association was not offset by an effect from the maternal genotype, as might have been expected through an effect of the same variants on maternal energy intake [19]. This association remained significant after adjustment for other possible confounders. These weight differences were rapidly attenuated after birth.

This is the first observation of such an inverse relationship between birth weight and FTO risk alleles for obesity in full-term, singleton, healthy newborns. An association between the more severe small-for-gestational age phenotype and risk alleles of FTO (odds ratio for SGA TA versus TT: 1.54; 95% CI: 1.07, 2.22], as well as for other risk alleles (PTER and KCNJ11, two high-risk alleles associated with obesity and diabetes in adults), has been found by Morgan et al. [20]. Other studies have also shown a similar link between the genetics of type 2 diabetes with low birth weight [21-25] or with small-for-gestational age [26,27]. In contrast, the first study to show an association between FTO and obesity [13], as well as other studies [12,28-31] and a meta-analysis by Kilpelainen et al. [32] (data from previous studies plus analyses of 4 large European birth cohorts), found no inverse association between the FTO risk allele and birth weight or evidence of a positive association by the postnatal age of 2 weeks (12]. In the meta-analysis by Kilpelainen et al. [32], among the 13 established risk alleles for obesity in various genes, only FTO (rs1121980) and MTCH2 (rs10838738) risk alleles were significantly associated with a high birth weight (+11 ± 4 g/allele; p = 0.013; n = 28,219) and low birth weight (−13 ± 5 g/allele; p = 0.012; n = 23,680), respectively. None of these associations remained significant after correction for multiple testing. Many factors may explain the discrepancy between these studies and our study. Different populations are exposed to different environmental and genetic influences that may interact with FTO variants. Some of the cohorts in the meta-analysis [32] were born a long time ago (between 1918 and 1975), and most had a birth weight ranging from 3361 to 3536 g, which is higher than that in our cohort and in newborns of European origin (3357 g) [33]. In some studies, data on birth weight were only self-reported, or reported by the participants’ mothers, or sometimes measured, but rounded to the nearest quarter of a pound. These conditions decrease the power to detect significant associations. Furthermore, most studies had no information on gestational age, maternal DNA, or other maternal/newborn characteristics that may confound the association. Finally, most previous studies did not exclude non-singleton births, individuals born preterm or post-term, gestational complications, or congenital malformations/perinatal problems. These factors are known to be associated with a greater prevalence of extreme birth weight.

Although replication in independent samples is essential, our finding is compatible with the hypothesis (also called the “fetal insulin hypothesis” [8]) that common genes that are inherited by the fetus affect birth size and predisposition to obesity, as well as its related complications in adult life [1-5]. How genetic variations in FTO contribute to variation in fetal weight may not be a simple explanation. In adults, the FTO gene is thought to contribute to weight gain by diminishing sensation of satiety and increasing energy and fat intake [34-36]. Such an explanation is not satisfactory in fetuses where the nutrients are completely provided by the maternal circulation. An indirect effect of the maternal FTO gene via a greater maternal energy intake is not conceivable because the maternal risk alleles were not independently associated with a low birth weight in our study. In the fetus, insulin is the main growth hormone and hyper- or hypoinsulinemia can lead to macrosomia or growth retardation, respectively [37,38]. Several studies have demonstrated a regulatory role of FTO on insulin secretion or sensitivity. In mice, induced expression of FTO enhances the first phase of glucose-induced insulin secretion in INS-1 cells of the pancreas [39]. In cultured human myotubes, FTO overexpression alters insulin signaling and increases *de novo* lipogenesis [40]. High-risk alleles of FTO are also associated with lower cerebrocortical insulin sensitivity [41]. The effect of FTO variants might also occur at the level of placenta where it is highly expressed [42], similar to many other tissues [9]. In animals, placental mRNA abundance of FTO is positively correlated with birth weight [43]. In humans, placental FTO expression is associated with increased fetal weight and length, and with placental weight in infants from non-primiparous women, as well as an increased fetal-to-placental weight ratio in primiparous women. There are also other intriguing findings regarding the possible effect of FTO in maternal-fetal interactions. In the ALSPAC cohort where maternal genotypes were available [44], a maternal “risk-allele score” (combining 4 risk alleles for obesity, including FTO rs9930609) was inversely associated with gestational weight gain in the first 18 weeks of pregnancy (214.46 g/wk per allele) compared with three other risk alleles for obesity. The maternal risk allele in FTO showed the greatest trend of a negative association with birth weight (−20.44 g; 95% CI: −42.65, 1.78; p = 0.07) [44]. Fetal FTO may participate either in the control of fetal weight gain or in the partitioning between maternal storage, placental development, and fetal growth. Interactions between maternal genetics and fetal metabolism or reciprocally have been previously demonstrated for lipoprotein metabolism [45,46]. However, how such interactions occur and an explanation for the inverse relation between the risk allele for obesity and low birth weight are still speculative at this stage.

We recognize that our study has some limitations. First, the associations observed in our study could be false positives. A false positive association is frequently caused by the confounding effect of population stratification when ethnicity or geographic origin is associated with the phenotype and genotype. We attempted to control for this type of bias by verifying and adjusting for the origin (especially Italian origin). Our sample size is small compared with the majority of genetic association studies. This resulted in a lower power to detect any associations with a high level of statistical significance. Calculation of statistical power using a mean difference per allele of 50 g showed that we had a 44% power to detect an association. Finally, birth weight is a simple measure that does not discern between fat mass and other components. Future studies need to investigate a more precise measure of fat mass in newborns using total body electric conductivity, dual energy x-ray absorptiometry, or air displacement plethysmography [47].

## Conclusions

In conclusion, the present study investigated 494 newborns with well-documented confounding factors that affect birth weight. After exclusion of pathological situations affecting birth weight, the FTO risk allele for obesity showed a significant, inverse association with birth weight. This association remained significant after correction for confounding factors and maternal FTO variants. This observation is compatible with the notion that genetic variants leading to obesity in later life may cause lower weight in fetal life, and supports a role for FTO in early growth.
